# Supplementary material for: Spatiotemporal relationships of coyotes and free-ranging domestic cats as indicators of conflict in Culver City, California
Source: PeerJ. 2022 Oct 7;10:e14169. doi: 10.7717/peerj.14169 (PMC9549883; doi:10.7717/peerj.14169)
Supplement: Supplemental Information 2 — (A) Ballona Creek. (B) Blanco Park. (C) Carlson Park. (D) Culver City Park. (E) Holy Cross Catholic Cemetery. (F) Inglewood Oil Fields. (G) Kenneth Hahn State Recreation Area. (H) Lindberg Park. (I) Syd Kronenthal Park. (J) Tellefson Park. (K) Veterans Memorial Park. Sources: Esri, Airbus DS, USGS, NGA, NASA, CGIAR, N Robinson, NCEAS, NLS, OS, NMA, Geodatastyrelsen, Rijkswaterstaat, GSA, Geoland, FEMA. [file peerj-10-14169-s002.pdf]

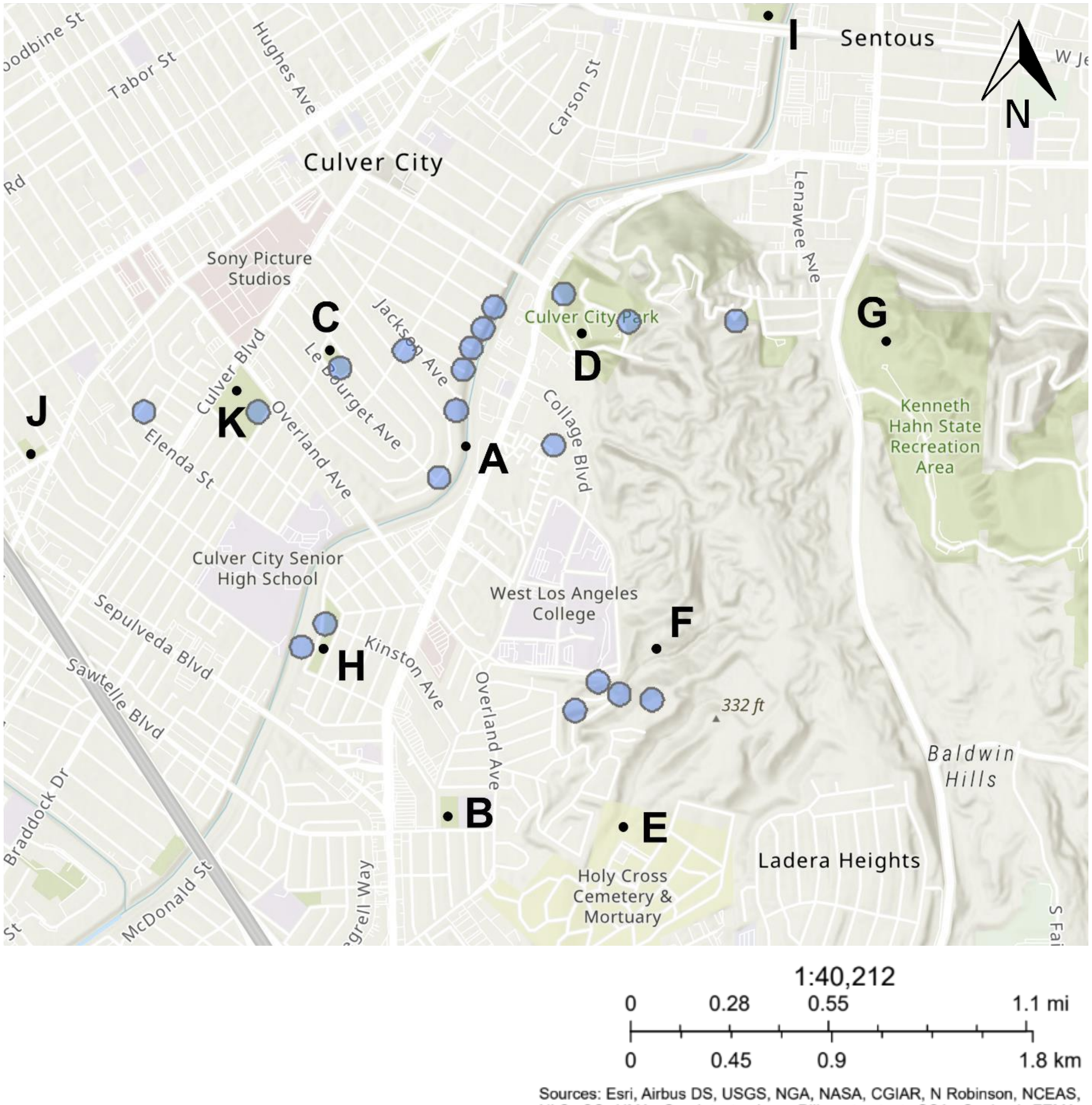

Sources: Esri, Airbus DS, USGS, NGA, NASA, CGIAR, N Robinson, NCEAS, NLS, OS, NMA, Geodatastyrelsen, Rijkswaterstaat, GSA, Geoland, FEMA,
