# Supplementary figures and images for: Spatiotemporal relationships of coyotes and free-ranging domestic cats as indicators of conflict in Culver City, California
Source: PeerJ. 2022 Oct 7;10:e14169. doi: 10.7717/peerj.14169 (PMC9549883; doi:10.7717/peerj.14169)

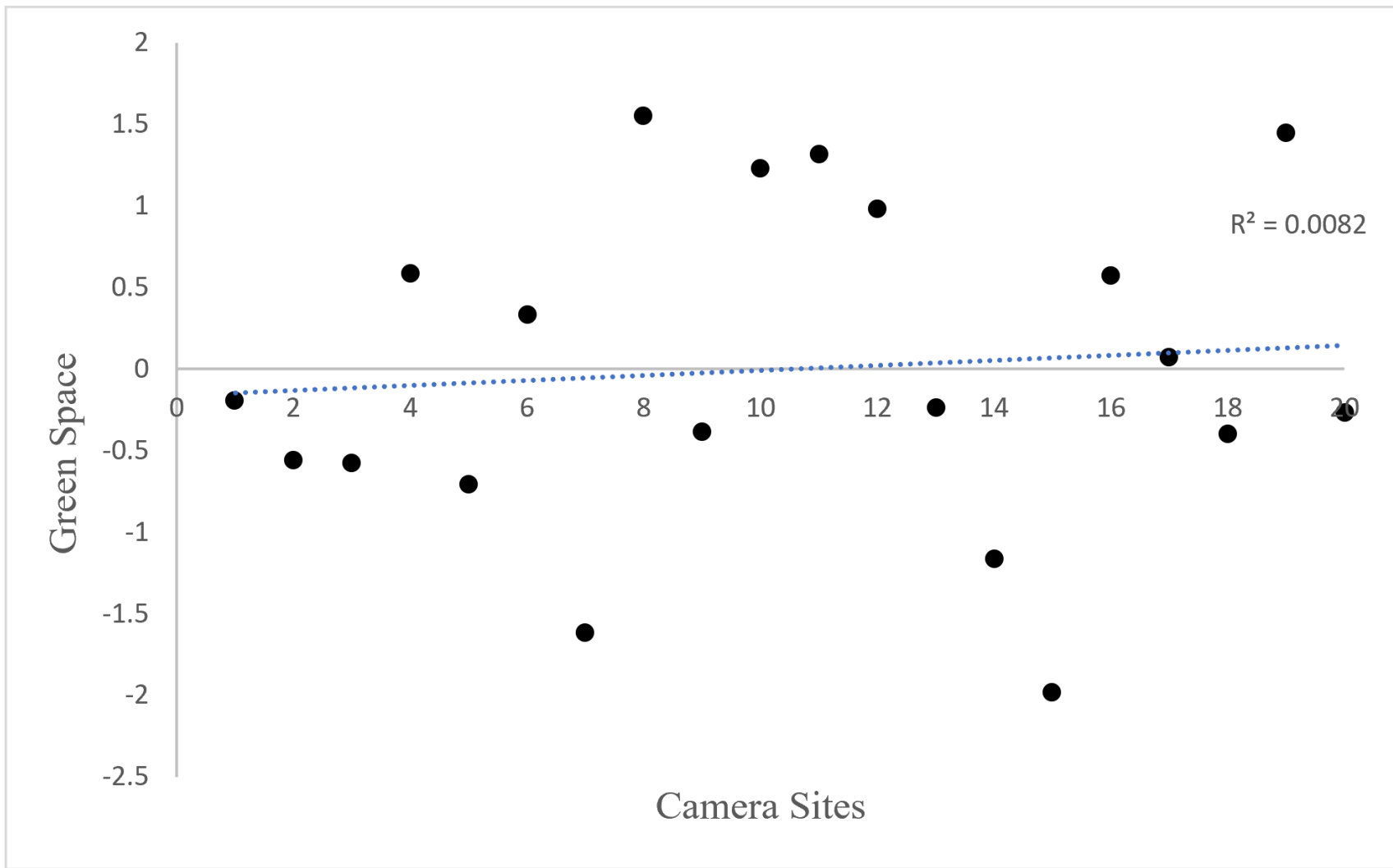

Supplement: Supplemental Information 3 — Nine sites have positive values of green space while eleven sites have negative values of green space. [file peerj-10-14169-s003.pdf]
