# Supplementary material for: Spatiotemporal relationships of coyotes and free-ranging domestic cats as indicators of conflict in Culver City, California
Source: PeerJ. 2022 Oct 7;10:e14169. doi: 10.7717/peerj.14169 (PMC9549883; doi:10.7717/peerj.14169)
Supplement: Supplemental Information 4 — Camera height, proportion of green space, and frequency of cat detections were standardized using z-scores. [file peerj-10-14169-s004.docx]

| Study area | Sampling nights | Camera height STND | Green space STND | Number of cats STND |
| --- | --- | --- | --- | --- |
| 1 – Jackson Ave | 200 | -1.2337 | -0.194 | 0.273965 |
| 2 – Carlson Park | 200 | -1.01663 | -0.56 | -0.51959 |
| 3 – Madison Ave | 200 | -0.43777 | -0.575 | 0.009447 |
| 4 – Blair Hills Park | 200 | 1.443541 | 0.584 | -0.44401 |
| 5 – La Salle Ave | 200 | 0.43053 | -0.707 | -0.55738 |
| 6 – Stocker St | 200 | 0.647604 | 0.334 | -0.4818 |
| 7 – Jasmine Ave Bike Path | 200 | 1.371183 | -1.616 | -0.55738 |
| 8 – Marycrest Manor | 200 | 0.43053 | 1.552 | -0.51959 |
| 9 – Veteran’s Park | 200 | -1.16135 | -0.384 | 0.047235 |
| 10 – Raintree Condos | 200 | -1.01663 | 1.229 | 0.122812 |
| 11 – Culver City Park1 | 200 | -0.43777 | 1.317 | 3.221451 |
| 12 – Culver City Park2 | 200 | -0.51012 | 0.98 | -0.51959 |
| 13 – Baldwin Ave | 200 | -0.7272 | -0.238 | -0.10392 |
| 14 – Marietta Ave | 132 | 0.719962 | -1.161 | 0.009447 |
| 15 – Flaxton St | 200 | -1.16135 | -1.983 | -0.40622 |
| 16 – Ballona Creek | 200 | 0.719962 | 0.569 | -0.33065 |
| 17 – Revere Pl | 95 | 0.358172 | 0.07 | 2.352321 |
| 18 – Jasmine and Le Bourget | 109 | -1.16135 | -0.399 | -0.55738 |
| 19 – Bridal Path | 200 | 1.877689 | 1.449 | -0.51959 |
| 20 – Lindberg Park | 200 | 0.864678 | -0.267 | -0.51959 |
