# Supplementary material for: Spatiotemporal relationships of coyotes and free-ranging domestic cats as indicators of conflict in Culver City, California
Source: PeerJ. 2022 Oct 7;10:e14169. doi: 10.7717/peerj.14169 (PMC9549883; doi:10.7717/peerj.14169)
Supplement: Supplemental Information 5 — NumRab, number of rabbits detected at each site. NumCat, number of cats detected at each site. [file peerj-10-14169-s005.docx]

|  | Green space | NumRab | Camera height | NumCat |
| --- | --- | --- | --- | --- |
| Green space | 1.000 | 0.623 | 0.180 | 0.240 |
| NumRab | 0.623 | 1.000 | -0.117 | 0.404 |
| Camera height | 0.180 | -0.117 | 1.000 | -0.155 |
| NumCat | 0.241 | 0.404 | -0.155 | 1.000 |
