# Supplementary material for: Spatiotemporal relationships of coyotes and free-ranging domestic cats as indicators of conflict in Culver City, California
Source: PeerJ. 2022 Oct 7;10:e14169. doi: 10.7717/peerj.14169 (PMC9549883; doi:10.7717/peerj.14169)
Supplement: Supplemental Information 6 — NumCat, number of cats detected at each site. [file peerj-10-14169-s006.docx]

| Covariate | VIV | Rank |
| --- | --- | --- |
| Greenspace | 0.973 | 1 |
| Camera height | 0.524 | 2 |
| NumCat | 0.205 | 3 |
